# Supplementary material for: Patterns of Intron Gain and Loss in Fungi
Source: PLoS Biol. 2004 Nov 30;2(12):e422. doi: 10.1371/journal.pbio.0020422 (PMC532390; doi:10.1371/journal.pbio.0020422)
Supplement: Table S1 — Also available at http://genes.mit.edu/NielsenEtAl/. (4.3 MB ZIP). [file pbio.0020422.st001.zip › NielsenEtAl/html/385.html]

AN3709.1.NCU06877.1.MG00871.1.FG07950.1


```
 CLUSTAL W (1.82) Multiple Sequence Alignments - Introns Inserted


Sequence 1: AN3709.1	445 aa
Sequence 2: FG07950.1	641 aa
Sequence 3: NCU06877.1	653 aa
Sequence 4: MG00871.1	479 aa
Alignment Length: 708 aa
Number Identitical Residues: 152 aa
Alignment Score (without introns) 8703


MG00871.1 	---------------~--------------------------------------------
NCU06877.1	----------MRLPP~PSTPTQLLRLRLTRSALSRSAVSTTSPLATSTCPSSQRTVSTKV
FG07950.1 	MSPVPTQYLLLISAW0TTTSIDLIFAAHVYARAIGFCRIFPQPIPSPMRAQLRRTICSHS
AN3709.1  	---------------~--------------------------------------------
          	                                                            

MG00871.1 	------------------------------------------------------------
NCU06877.1	G----AATITPGLRFQSLLFPPSTGITTTTTTTTTAFFSRNNLSTSTTTVNKRDQTVKPT
FG07950.1 	PSVRSSQSLTRHIQLRSSVLSSSLLRLPLNKPRATPTLTRSLATARYLTSPRIRPTTLKT
AN3709.1  	------------------------------------------------------------
          	                                                            

MG00871.1 	------------------------------------------------------------
NCU06877.1	VTLAPSTPVVLRASVTGLGLLFVLGLGLGYG--ITGFLGQKSTEQQELPSEASYTTIQNN
FG07950.1 	LDCQTVVAKRSHITASGAAGFSSSGNPPLQANLLHSVVIAAAVIATAVAVSAWTVGSSNN
AN3709.1  	------------------------------------------------------------
          	                                                            

MG00871.1 	--------------MPEIPPGRPGNLTPEQDELLRKLWSRLFEVCGVIQP------EAPA
NCU06877.1	IQDILDDESSTTNMSREIAPGRPGNLTPEQEEKLKQLWKLIFQVCGVSSPSPSSESSTPA
FG07950.1 	PPPPGDIEEEIAIMSFQVPPGRPGNLTPEQEEKLRKLWVAVFQLTGVADEE--SSGAEIL
AN3709.1  	-------------MPIEAAPGTVGNLTPEQEAKLQEFWVLLFKVCGVQMDG--IEETENR
          	                : .**  *******:  *:::*  :*:: **       .     

MG00871.1 	RAPETADADADSTKKKTRTGFFSRRK---------TDASTTNG------------PTQEG
NCU06877.1	ATSDDVSIKGDDKKKKSKLSMFSRKHKKDSDDTTPDSASTASGSMPGSPAIVLNASTTSG
FG07950.1 	SPKEEISSADADQKKKRGFGMFKKGK-------------SGTST-----------PTESA
AN3709.1  	PPS---PAQEKKQPPKRRFGFFGGGSQEKDESTAANDAASGIAS-----------LTITD
          	 .         .   *   .:*    ......::. .:::  .:            *   

MG00871.1 	DPEDKYGQNKIFQDTLANMPPDEIRKTIWSMVKFDHPDALVLRFLRARKWDVEKALVMLV
NCU06877.1	D-DDKYNQLSKFHETLASQSPAAIRETIWSMVKHDHPDALVLRFLRARKWDVEKALVMFI
FG07950.1 	D-EDKYNETKQFHETLAKETPETIRHTIWSMVKHDHPDALVLRFLRARKWDVEKALVMLV
AN3709.1  	G-DDKFGTSKEFQKALAEIKPEDMRTAFWSMVKQDNPDSLLLRFLRARKWDVKKALMMLI
          	. :**:.  . *:.:**.  *  :* ::***** *:**:*:***********:***:*::

MG00871.1 	SSMYWRCKEVKVDSNIMSRGEGGMSQDEKEAPEGSDE---QKMGKGFMAQLRMGKSLAHG
NCU06877.1	STMNWRDNEMKVDADIMRSGEGGAAEAEKMEAKTSEEIAKKKLAIDFLTQTRMGKSYVHG
FG07950.1 	ATMNWRHNDMKVDDDIMKNGDAFAVEDEK-TDSATKQ-----VSADMMKQLRMGKSFLHG
AN3709.1  	STIRWRLLDAKVDEDIMVNGEHLALEQLKSSDSAERK-----KGEDFIKQFRLGKSFLHG
          	::: **  : *** :**  *:    :  *   .   :      . .:: * *:***  **

MG00871.1 	EDKLGRPVVYVKVRLHKAADQTPESIERYTIFLIETTRALIKPPIDTA0TLVFDMTDFSM
NCU06877.1	VDKQGRPICFVRVRLHKQGEQSEESLERYTVYLIETCRMLLQGSVDTA0TIVFDMTGFSM
FG07950.1 	TDKQGRPICVVRVRLHKAGQECEESLEKYTVYIIETARMTLEPPVDTA0CIVFDMTGFSM
AN3709.1  	VDKLGRPICYVRVRLHRAGDQDIEALDRFTVFTIESARMMLVPPVETA0-----------
          	 ** ***:  *:****: .::  *:::::*:: **: *  :  .::**            

MG00871.1 	ANM0DYSPVKFMIKCFEANYPESLGAVVVHKAPWVFQG1IWRIIRGWLDPVVAAKVNFTN
NCU06877.1	ANM~DYAPVKFMVKCFEANYPECLGAVLVHKAPWIFQG1IWRVIRGWLDPVVANKVHFTN
FG07950.1 	ANM~DYTPVKFMIKCFEANYPESLGAVLVHKAPWLFQG1IWKVIRGWLDPVVAAKVHFTN
AN3709.1  	---~DYHPVKFMIKCFEANYPESLGVVLIHKAPWIFSS1IWNVIKGWLDPVVAAKIQFTK
          	    ** *****:*********.**.*::*****:*.. **.:*:******** *::**:

MG00871.1 	DITALQEYVSIDKIPVELEGKEQWEYKYTEPIPGENDRMKDTATRDRLLAERAKLYTAFE
NCU06877.1	NISEMSEFISPEKLPKDLDGQEDWEYKYVEPVPGENDRMKDEETISKLQAARAELVKEYE
FG07950.1 	NRSELEDFIAPNHLIKELEGDENWAYKYVEPSPGENDKMKDTQTRDRLLVEREELVKKFE
AN3709.1  	TQQDLEEFIPKSRIITELEGDEKWEYKYIEPKEGENDKLKETAKRDELITQRQKLAKEVQ
          	    :.:::. .::  :*:*.*.* *** **  ****::*:  . ..* . * :* .  :

MG00871.1 	EATLKMLRNP------TDQAVRKERSEIASTLRSQHWQLDPYIRARSWYDRTGVLREDGT
NCU06877.1	EATLQWIQASG-----NSEELKKKRDAIAEKLRVDYWNLDPYVRARSFYDRIGVLLPGGK
FG07950.1 	QATREWIRHP---EGEQGKQIKSEREKIAKLLKEDYWHLDPYIRARTLYDRQGAIQSGGK
AN3709.1  	DATVAWILASRKKEEDKAKEVTEKRKDLIGRLRTQYWQLDPYVRAPSLYDRLNIIQGDGK
          	:**   :  . ... .  : : .:*. :   *: ::*:****:** : *** . :  .*.

MG00871.1 	INYYPPKDFKYVVNGGGQQKQED~NGIKKVDSAHEKASIQDSAPPAVSLNGTA
NCU06877.1	LDPFLE------------GKKDG0LAVRVVGV---------------------
FG07950.1 	TDWYSLK------------PPTA~AASTSADDLD-------------------
AN3709.1  	IEFYPEA------------ATNG~EAAKSN-----------------------
          	 : :                     .
```
